# Impact of Preoperative CT-Diagnosed Sarcopenic Obesity on Outcomes After Radical Cystectomy for Bladder Cancer

**DOI:** 10.3390/cancers17162669

**Published:** 2025-08-15

**Authors:** Alberto Artiles Medina, Mariam Bajawi Carretero, Enrique López Pérez, Sara Garach Fernández, David López Curtis, Leyre Elías Pascual, José Daniel Subiela, Javier Soto Pérez-Olivares, Catalina Nieto Góngora, Fernando González Tello, Irene de la Parra Sánchez, César Mínguez Ojeda, Victoria Gómez Dos Santos, Francisco Javier Burgos Revilla

**Affiliations:** 1 Urology Department, Ramón y Cajal University Hospital, University of Alcalá, 28034 Madrid, Spain; 2 Department of General Surgery, University Hospital of Guadalajara, 19002 Guadalajara, Spain; 3 Biomedical Engineering, Universidad Politécnica de Madrid (UPM), 28034 Madrid, Spain; 4 Engineering & Innovation Unit, Radiology Department, Ramón y Cajal University Hospital, 28034 Madrid, Spain; 5 Radiology Department, Ramón y Cajal University Hospital, 28034 Madrid, Spain

**Keywords:** body composition, sarcopenia, obesity, sarcopenic obesity, cystectomy, bladder cancer

## Abstract

Objective: To evaluate the impact of body composition parameters, including specifically sarcopenic obesity (SO), on postoperative and oncological outcomes in patients undergoing radical cystectomy (RC) for bladder cancer, thereby addressing a paucity of data in this setting. Methods: A retrospective observational study was conducted in patients who underwent RC. Preoperative CT scans were analyzed using semi-automatic segmentation software to assess body composition parameters, with measurements of adipose and muscle tissue obtained at the level of the L3 vertebra. Results: A total of 249 patients were included, of whom 127 (52.5%) met the criteria for sarcopenia, 53 (21.3%) for obesity, and 14 (5.6%) for SO. Multivariate analysis identified previous abdominal surgery (OR 2.56, 95% CI 1.24–5.23, *p* = 0.011), total serum protein level (OR 0.57, 95% CI 0.36–0.88, *p* = 0.013), and SO (OR 7.01, 95% CI 1.06–37.05, *p* = 0.045) as independent predictors of 90-day postoperative complications. Patients with SO experienced significantly higher rates of abdominal wall complications (*p* = 0.03). However, in multivariate analyses, SO was not associated with overall survival (despite a *p* value of 0.04 at univariate analysis), cancer-specific survival, or progression-free survival. Conclusions: Preoperative CT-based assessment of body composition is a valuable tool in the surgical evaluation of patients undergoing RC. SO appears to be an independent predictor of short-term postoperative complications and should be considered when planning prehabilitation strategies.

## Simple Summary

Sarcopenia has been widely investigated as a predictor of postoperative complications. More recently, increasing attention has been devoted to additional body composition parameters, such as sarcopenic obesity. This study contributes to the growing body of evidence supporting the role of body composition assessment in predicting outcomes after radical cystectomy. Our findings emphasize the clinical utility of preoperative computed tomography (CT)-based body composition assessment, particularly highlighting sarcopenic obesity as a significant predictor of 90-day postoperative complications. However, despite its value in identifying patients at higher risk for complications, CT body composition assessment did not demonstrate a significant impact on survival in this study.

## 1. Introduction

Body composition assessment, defined as measurement of the proportions and distribution of adipose tissue and skeletal muscle, is becoming increasingly popular for the purposes of disease prognosis and treatment planning. Poorer clinical outcomes have been noted in several groups of patients, especially including cancer patients, in the presence of certain abnormal body composition features; these include, but are not limited to, derangements in skeletal muscle mass, visceral adiposity, and subcutaneous adiposity [1].

Sarcopenia, obesity, and sarcopenic obesity (SO) are regarded as syndromes of disordered body composition within a spectrum of relative muscle mass and percentage of body fat [2]. Baumgartner et al. [3] first proposed criteria for SO in 2004; however, there is considerable heterogeneity in the methods currently used to define SO in the literature [2], hindering data synthesis and comparison between studies [4].

Sarcopenia has been widely studied and proven to be associated with poor outcomes in cancer patients. Nevertheless, the impact of SO, a condition involving a combination of sarcopenia and obesity burden, has been investigated less frequently, particularly in the field of bladder cancer [4].

SO has recently emerged as a novel and promising point of focus in body composition assessment for prediction of clinical outcomes in various malignancies. The reported overall prevalence of SO among cancer patients varies substantially, from 1% to 29% [4]. Various negative impacts of SO on clinical outcomes in cancer patients have been documented, including a higher risk of dose-limiting toxicity; a higher rate of surgical complications; and a shorter survival [4]. Growing evidence suggests that there is a relationship between SO and survival outcomes in various cancers, although some other authors have reported no significant association [2].

Interestingly, SO has been associated with unfavorable outcomes in a variety of malignancies. Specifically, in esophago-gastric cancer patients undergoing radical resection, SO (prevalence 5.7–28.7%) is associated with a worse overall survival (OS) and a higher postoperative complication rate [5]. Similarly, among patients undergoing surgery for colorectal cancer, those with SO (prevalence 14.5%) have a higher overall rate of complications and an increased risk of 30-day mortality [6]. In patients with primary liver cancer, SO (prevalence 6.67–30%) is correlated with a poor OS and recurrence-free survival [7]. Patients with pancreatic cancer and SO (prevalence: 0.6–25%) likewise display poorer overall survival and worse postoperative complications [8], and in prostate cancer patients, SO (prevalence: 24%) contributes to a higher non-cancer mortality [9]. Finally, a recent study in a cohort of patients with solid tumors showed that SO is significantly associated with lower OS, poorer quality of life, and higher risk of intensive care unit (ICU) admission; the prevalence of SO was 4.36% in this cohort according to European Society for Clinical Nutrition and Metabolism (ESPEN) criteria [10].

Furthermore, to date, most research in the context of radical cystectomy (RC) has centered on sarcopenia alone, with the reported prevalence varying widely due to the use of heterogeneous definitions and assessment criteria. Despite this variability, multiple studies have linked sarcopenia to adverse postoperative outcomes, including increased complication rates and decreased survival [11]. Only a limited number of studies have specifically addressed the association between SO and clinical outcomes in bladder cancer (BC). A recent systematic review found that 31.3% of patients undergoing RC for BC had sarcopenia [12], while other studies have reported SO in 3.6–27% [13,14,15].

As there is a paucity of published data relating to CT-measured body composition parameters, especially SO, in patients undergoing RC for BC and their influence on outcomes after surgery, this study aimed to analyze the impact of preoperative CT body composition, particularly SO, on surgical and oncological outcomes after RC for BC.

## 2. Methods

### 2.1. Patients and CT Scan Information

A retrospective observational study of patients undergoing RC for BC between January 2012 and December 2019 was conducted. Preoperative CT scans (within 90 days before surgery) were obtained from all patients. Measurements of adipose tissue, as well as muscle tissue, were performed on a cross-section of a preoperative contrast-enhanced CT scan at the level of the third lumbar vertebra (L3), bearing in mind that quantitative measurements of the cross-sectional skeletal muscle area (SMA) and SMI (SMA/height^2^) using CT imaging are most commonly assessed at this level [16], given its strong correlation with whole-body musculature (r = 0.86). Also, this method is considered the gold standard for diagnosis of low muscle mass and sarcopenia [17]. Only patients with available CT scan and postoperative follow-up in our center were included. To reduce potential bias due to the exclusion of patients with early adverse outcomes, no minimum follow-up time was required. Patients with missing information regarding main outcomes or patient’s height were excluded. Semi-automatic segmentation using the Synapse 3D V6.7 (Fujifilm) [18] software was used to determine various parameters of body composition on the CT scan. 

Several body composition parameters were retrieved from a single CT slice image, including total muscle area (TMA), visceral fat area (VFA), subcutaneous fat area (SFA), total fat area (TFA), skeletal muscle index (SMI), psoas muscle index (PMI), and subcutaneous adipose tissue index (SATI) (SATI = SFA/m^2^). CT images were segmented by two well-trained clinical experts and one biomedical engineer supervised by a software expert. Supplementary Figure S1 illustrates the steps for CT segmentation and body composition assessment.

**Figure 1:**
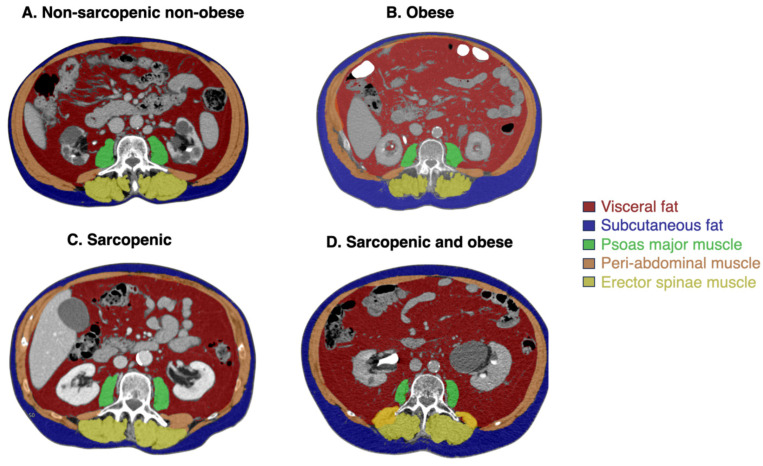
Images showing different body composition phenotypes. Axial CT slides showing (**A**) non-sarcopenic and non-obese patient with BMI 24.91 kg/m^2^, SMI 54.93 cm/m^2^, and VFA 149 cm^2^; (**B**) obese patient with BMI 36.05 kg/m^2^, SMI 60.46 cm/m^2^, and VFA 340.31 cm^2^; (**C**) sarcopenic patient with BMI 18 kg/m^2^, SMI 35.33 cm/m^2^, and VFA 96 cm^2^; and (**D**) sarcopenic and obese patient with BMI 30.85 kg/m^2^, SMI 51.7 cm/m^2^, and VFA 393.33 cm^2^.

Skeletal muscle mass index (SMI) (sum of the area of the musculature at that level (cm^2^) and normalized by the square of the patient’s height (m^2^)) was calculated. Sarcopenia, obesity, and SO were calculated according to sex-specific cut-off points according to international definitions (Prado et al.’s definition [19] and the later modification by Martin et al. [20]). SO was defined as present when the criteria proposed by Prado et al. [19] were met, corresponding to obesity (BMI ≥ 30 kg/m^2^) + sarcopenia (SMI L3 ≤ 52.4 cm^2^/m^2^ in men and ≤38.5 cm^2^/m^2^ in women). Figure 1 presents images showing different body composition phenotypes.

### 2.2. Variables

Baseline [age, sex, body mass index (BMI)], clinical (ASA, tumor features, preoperative comorbidities), laboratory [neutrophil-to-lymphocyte ratio (NLR), creatinine, cholesterol], surgical (approach, technique), and outcome data (number and type of complications and overall, cancer-specific, and progression-free survival) were collected. Overall survival (OS) was calculated from the day of RC to the time of death or final follow-up (in months). Progression-free survival (PFS) was defined as the time from RC to tumor progression or death by any cause.

### 2.3. Statistical Analysis

Continuous variables were expressed as mean values with standard deviations for normally distributed data, or as median values with first and third quartiles. Categorical variables were expressed as absolute and relative frequencies. Differences between variable groups (SO and non-SO groups) regarding types of complications were assessed using the χ^2^ test or Fisher’s exact test when any cell contained less than five cases. Univariate and multivariate logistic regression analyses were applied to identify variables significantly related to postoperative complications (main outcome 1). Overall survival (OS) (main outcome 2), cancer-specific survival (CSS) (main outcome 3), and progression-free survival (PFS) (main outcome 4) were estimated by Kaplan–Meier curves. Cox proportional hazards models for estimating the effects of covariates on the hazard of the occurrence of the survival event were employed. Variables with *p* < 0.2 in univariate analysis and those of known clinical relevance were included for further multivariate analysis. The number of covariates tested by the Cox method and logistic regression accounted for the number of patients with the event of interest. Final models were obtained using backward stepwise selection. A two-tailed *p* value of less than 0.05 was considered statistically significant. All data analyses were performed using the STATA 18.0 (StataCorp, College Station, TX, USA).

## 3. Results

A total of 249 patients were included. The baseline characteristics of the study population are shown in Table 1. The mean age was 73.89 (SD 10.03) years, and there was a predominance of males (81.50%). Most patients were ASA class II (57.26%). Seventy-two patients (28.92%) had a history of previous rectal or abdominal surgery. The laparoscopic approach was used in 116 (46.77%) patients, and the ileal conduit was the most commonly used type of urinary diversion (80.93%). The mean BMI was 27.49 (SD 4.16) kg/m^2^. The BMI categories were 2.01% underweight (5/249), 29.72% normal weight (74/249), 46.99% overweight (117/249), 15.26% obesity class 1 (38/249), 4.82% obesity class 2 (12/249), and 1.2% obesity class 3 (3/249).

Of the 249 patients, 127 (52.48%), 53 (21.29%) and 14 (5.6%) met the criteria for sarcopenia, obesity, and SO, respectively. Mean SMI was 48.89 (42.47–54.36) cm^2^/m^2^. The mean PMI was 5.80 (4.76–7.17) cm^2^/m^2^. Supplementary Table S1 shows the relationship between body composition phenotypes and tumor stage, lymph node involvement (N+), and history of neoadjuvant chemotherapy (NAC). Although not statistically significant, the number of sarcopenic patients appeared to be higher in the N+ and NAC groups. 

The mean serum protein level among patients who underwent RC was 6.65 (SD 0.80) g/dL. Overall, the 90-day mortality was 2.83% in our series. The 90-day complication rate was 60.24%, with 46/150 (30.66%) complications being Clavien-Dindo 3 or greater. Median follow-up was 32.46 (16.43–53.1) months. The relative frequency of overall complications was 85.71% in the 14 patients with SO (12/14) and 57.69% in the remaining non-SO patients (*p* = 0.04). Supplementary Figure S2 presents a bar graph showing the incidence of postoperative complications in the groups with and without SO.

Table 2 shows the results of univariate and multivariate analyses for different possible predictive factors of postoperative complications within 90 days after RC. Prior rectal or abdominal surgery (OR 2.56, 95% CI 1.24–5.23, *p* = 0.011), serum total protein level (OR 0.57, 95% CI 0.36–0.88, *p* = 0.013), and SO (OR 7.01, 95% CI 1.06–37.05, *p* = 0.045) were all identified as independent predictors of complications by multivariate analyses. Table 3 shows the results of tests for the association between SO and different types of postoperative complications. Patients in the SO group had significantly higher abdominal wall-related complications than those without SO (15.38% vs. 3.43%, *p* = 0.036). No significant differences in the rates of bowel fistula, urinary leakage, or overall intra-abdominal complications were observed between the groups.

Table 4 displays the results of the uni- and multivariate Cox proportional hazard models evaluating the potential prognostic value of various parameters (including SO) for OS, CSS, and PFS. Univariate analysis revealed SATI (HR 0.99, 95% CI 0.98–0.99, *p* = 0.03) and SO (HR 2.01, 95% CI 1.01–3.99, *p* = 0.04), among others, to be of prognostic significance for overall survival. However, these parameters were not statistically significant predictors of OS, CSS, and PFS in multivariate analyses. Multivariate analyses demonstrated that the prognostic factors for OS after RC were age, lymphovascular invasion (LVI) on RC specimen, stage > T1, and N+; for CSS, these factors were age, prior LVI, carcinoma in situ on RC specimen, LVI on RC specimen, and N+; and finally, for PFS, the predictors were age, stage > T1, and N+. Supplementary Figure S3A–C show Kaplan–Meier survival curves stratified by the presence of SO in the entire cohort for OS (A), CSS (B), and PFS (C). Although there appeared to be evidence of a trend in the influence of SO on OS, it was not a significant predictor at multivariate Cox analysis.

## 4. Discussion

This study adds to the existing body of literature on the subject of body composition parameters as predictors of outcomes following RC. In particular, it highlights the relevance and usefulness of CT body composition assessment before RC, as it can predict postoperative complications. While SO was found to be a predictive factor of 90-day postoperative complications, no significant influence on OS, CSS, or PFS was revealed.

The exact mechanism linking SO and poor outcomes in terms of postoperative complications remains unclear. Some pathophysiological aspects of SO, including chronic low-grade inflammation, impaired immune response, and decreased functional reserve, could offer a plausible explanation for this link. The inflammatory cytokines produced by adipose tissue, especially visceral fat, accelerate muscle catabolism, thereby contributing to the vicious cycle that initiates and sustains SO. Moreover, this process is associated with insulin resistance and dysmetabolism. All of these factors may amplify the physiological stress response to major surgery and chemotherapy, resulting in increased complications [21].

The contrasting lack of a significant association between SO and long-term survival outcomes may reflect the predominant influence of other oncological factors—such as tumor stage, pathological features, and response to adjuvant therapy—on these outcomes. However, outside of an oncological context, SO represents a major health concern as it is associated with frailty and disabilities, including cardiovascular disease and cancer, as well as increased all-cause mortality; these associations are attributable to the underlying deleterious biological mechanisms involved in SO, such as insulin resistance, lipotoxicity, mitochondrial dysfunction, oxidative stress, chronic inflammation, and proteostasis [22].

Body composition parameters other than sarcopenia remain underexplored in the field of bladder cancer and RC. Supplementary Table S2 summarizes the studies reporting data on the predictive value of sarcopenia and SO with respect to postoperative outcomes (complications and survival) following RC ([13,14,15,23,24,25,26,27,28,29,30,31,32,33,34,35]). The majority of the studies revealed worse survival outcomes in sarcopenic patients after RC, although there was considerable inconsistency in the results concerning postoperative complications.

The numerous proposed definitions of sarcopenia and SO pose a challenge in elucidating the role of these parameters in postoperative outcomes after RC. Several CT-derived cut-off values for SMI, with considerable variability depending on BMI and population characteristics, have been reported in the literature [19,20,36,37]. Prado et al. [19] initially established SMI thresholds in a cohort of obese patients (BMI ≥ 30 kg/m^2^) with gastrointestinal or respiratory tract malignancies. This dataset was later expanded by Martin et al. [20], who provided reference values for individuals with normal weight and overweight, using optimal stratification based on mortality risk. These thresholds have been widely adopted in subsequent studies despite limitations regarding their generalizability. Only one additional study has proposed BMI-specific reference values for skeletal muscle mass [36]. While obesity is most commonly defined by BMI (≥30 kg/m^2^), other studies have incorporated alternative metrics such as percent fat mass (%FM), visceral fat area (VFA), or fat-to-muscle ratios, including VFA-to-total abdominal muscle area (TAMA) ratio. This methodological heterogeneity underscores the lack of consensus on standardized definitions for body composition abnormalities, complicating cross-study comparisons and highlighting the need for population- and disease-specific validation of such thresholds [38]. In this study, we employed the definition of SO proposed by Prado et al. [19] (with inclusion of BMI criteria for obesity) because it represents one of the most extended definitions across studies of body composition.

The rate of sarcopenia reported in different studies ranges from 30.9% [14] to 68.8% [24] for patients undergoing RC, revealing great variability. In our series, 52.48% of patients exhibited sarcopenia. The prevalence of SO has been reported in only three studies. In a multicenter series by Mayr et al. [13], this condition was identified in 18 patients (3.6%), whereas a higher rate was reported by Psutka et al. [15] (16.4%). In our study, 5.6% of patients were classified as sarcopenic and obese.

Obtaining body composition variables derived from the preoperative CT scan is easy and can help identify patients at higher risk for postoperative complications. Indeed, automatic CT image segmentation provides similar results to manual measurements of body composition [39]. Recent technological advances have enhanced the use of body composition assessment, mainly due to artificial intelligence (AI) automation, which has reduced the analysis time from 15 to 20 min to mere seconds. These innovations effectively address previous implementation barriers and enable practical clinical application with minimal resource demands, creating opportunities for targeted interventions and personalized care pathways. In this study, we used a pragmatic and sophisticated semi-automatic software that made hardly any mistakes in delimiting the muscle area and visceral fat. These variables could be routinely included in radiology reports of preoperative CT scans from patients in whom RC is planned.

Many risk factors for postoperative complications after RC have been described. For example, prior abdominal surgery, extravesical disease, and prior radiotherapy are well recognized predictive factors in this setting [40]. Furthermore, low preoperative serum albumin has been linked with wound-related and gastrointestinal complications after RC [41,42]. In this regard, a systematic review by Ornaghi et al. [43] concluded that high BMI, hypoalbuminemia, and sarcopenia significantly increase the complication rate after RC. In addition, hypoalbuminemia was found to impact negatively on 3-year OS, and sarcopenia had similar effects on 5-year CSS and OS. The authors advocated the preoperative assessment of RC patients’ nutritional status as a valuable tool for predicting perioperative and survival outcomes. In our series, prior rectal or abdominal surgery, serum total protein level, and SO were proven to be predictors of complications. The first two factors are consistent with what has been previously published in this context. SO could be of additional value for risk stratification of patients undergoing RC for BC, though more research is required to further evaluate the role of this factor in this setting.

It has been reported that SO may create a synergistic effect whereby sarcopenia and obesity amplify each other [44]. Thus, we separately evaluated sarcopenia, obesity, and the combination thereof, treating SO as a distinct and potentially clinically valuable factor. Indeed, at multivariate analysis, SO was found to be the sole body composition parameter that remained an independent predictive factor for postoperative complications at 90 days.

The presence of sarcopenia among patients with BC undergoing RC is a well-researched factor associated with adverse survival outcomes and postoperative complications [45]. As pointed out above, in the present study, the number of sarcopenic patients appeared to be higher in the N+ and NAC groups, although the difference did not reach statistical significance (see Supplementary Table S1). Prior research has revealed that in patients who receive NAC, significant changes in body composition are observed during chemotherapy, with loss of muscle mass leading to an increased incidence of presurgical sarcopenia [46,47]. With regard to the tendency observed among patients with lymph node involvement (N+), it is worth mentioning that some features of cancer cachexia, such as anorexia and systemic inflammation, can contribute to sarcopenia [48].

Looking at Supplementary Table S2, data from the scarce available studies regarding the influence of sarcopenia on survival outcomes are conflicting, although most of these studies seem to point in the same direction: a relationship between sarcopenia and poor survival outcomes. It is worth mentioning the multicenter study by Mayr et al. [13], which showed sarcopenia to be an independent predictor of OS and CSS. Apart from Mayr et al.’s study, only the study by Psutka et al. [15] has reported data on this topic, and these authors reported that SO did not independently predict cancer-specific mortality or all-cause mortality. Similarly, our results indicate that SO is not a statistically significant predictor of OS, CSS, or PFS.

Stangl-Kremser et al. [2] systematically reviewed the literature on the prevalence and prognostic impact of SO in genitourinary cancer, notably for patients with renal cell carcinoma (RCC), urothelial carcinoma (UC), and prostate cancer undergoing treatment. They noted a real lack of studies addressing this topic in the field of urology (overall, only 15 papers: 5 for prostate cancer, 4 for RCC, 5 for BC, and 1 for upper urinary tract UC). They also estimated that the mean prevalence of SO in urological cancers was 27%. Only one out of ten studies showed a significant association of SO with OS, while another reported a trend but did not reach statistical significance. An additional paper identified SO as an adverse prognostic factor for CSS. The remaining studies did not demonstrate SO to be of prognostic relevance with regard to OS, CSS, or PFS. These prior results support the conclusions of the present study.

Focusing on the scarce studies approaching the importance of SO in predicting different outcomes of UC, a previous study by Kocher et al. [49] (*n* = 100) investigated the impact of SO on perioperative and cancer outcomes in patients with upper urinary tract UC undergoing radical nephroureterectomy and concluded that this biomarker is associated with non-BC disease relapse. Another study evaluated the prognostic significance of several body composition and nutritional parameters on outcomes for BC patients who were unfit for RC or systemic chemotherapy and were treated with radiotherapy. Of all the analyzed parameters, SO was associated with CSS, and a low prognostic nutritional index was associated with OS in this setting [50]. Additionally, a recent study (2024) has suggested that SO may help predict tolerance of NAC in patients with non-metastatic BC receiving this treatment prior to RC [51].

Beyond sarcopenia and SO, other parameters of body composition have hardly been investigated to date in BC patients. A recent study by Sharma et al. [35], however, paves the way for the use of AI for the assessment of body composition parameters in patients with RC and thus represents the start of a process whereby this approach could soon be fully integrated into routine clinical practice. This study showed that automated AI body composition measurements obtained preoperatively display a correlation with post-RC complications. According to the authors, an increase in subcutaneous adipose tissue area leads to more wound complications, while patients with increasing visceral adipose tissue area are more likely to experience infectious-related complications. Furthermore, every 10 cm^2^ increase in fat mass index is associated with more infectious and wound complications. This study also confirmed that higher SMI is associated with lower odds of major complications. In contrast, in the present study, CT measurements of fat tissue, i.e., visceral fat area (VFA), subcutaneous fat area (SFA), and total fat area (TFA), were not associated with post-RC outcomes.

According to Liu et al., SO is associated with poor OS in patients with cancer and should be integrated into the clinical care of these patients [10]. As previously mentioned, this parameter has become popular for the purpose of prediction of complications in systemic and surgical treatment in various cancer types. Several strategies could be used to minimize the detrimental effects of SO, e.g., the incorporation of some measures into the Enhanced Recovery After Surgery (ERAS) protocols, such as more exercise programs containing strength and aerobic exercise in combination with dietary interventions including a supervised weight loss program and/or protein supplements [52]. While some specific strategies for SO patients have been proposed in the literature, trials are needed to evaluate the outcomes of these strategies. Regarding nutritional aspects of prehabilitation in SO patients, protein prescription of 1.3 g/kg/day is recommended for muscle preservation, as well as a very low-calorie, high-protein formula [53]. In addition, prehabilitation programs also integrate a range of exercises focusing on both aerobic and resistance training. The duration and intensity of aerobic exercises should be personalized. However, a common goal is to achieve approximately 150 min of moderate aerobic activity per week, spread over 3–5 sessions lasting 30–50 min each [54]. Emerging evidence supports the potential reversibility of SO through prehabilitation in surgical oncology patients. For instance, in esophageal cancer, high adherence to exercise led to smaller declines in skeletal muscle index and reductions in visceral fat [55], while adequate protein intake improved muscle density [56]. Furthermore, patients referred for prehabilitation showed significant functional improvements despite baseline SO, highlighting the efficacy of targeted interventions [57].

To the best of our knowledge, this research presents the first analysis of the influence of CT-measured SO on outcomes after RC, emphasizing its role in 90-day complication prediction. Another strong point of our study is the performance of an analysis that goes beyond sarcopenia and considers other body composition parameters. The most important limitations lie in the retrospective nature of the study and the existence of some missing values in our dataset (some patients were excluded because of unavailable CT scans). Other limitations include the relatively small sample size and the potential presence of unmeasured confounders. Because of the small number of cases of SO in our series and the wide confidence interval for the association between this condition and postoperative complications, these findings warrant further research to externally validate and generalize the observed associations in larger, independent cohorts. Moreover, some factors could have influenced the accuracy of body composition assessment, and especially assessment of the skeletal muscle area; such factors include variability in CT slice selection and the timing of imaging acquisition. In our study, the interval between the CT scan and RC ranged from 0 to 90 days. Additionally, some patients underwent NAC, which, as already discussed, could have influenced body composition parameters. The impact of SO on OS was only observed at univariate analysis. Future studies with sufficient statistical power may better clarify the role of SO in predicting survival outcomes of RC patients.

## 5. Conclusions

The analysis of CT-measured body composition before RC provides information relevant to the prediction and prevention of postoperative complications, and such information could be utilized for patient counseling. CT-diagnosed SO is associated with postoperative complications but does not appear to influence long-term survival following RC. These results should be interpreted in the context of limitations including retrospective collection of data, a relatively small sample size, and absence of universal definitions for SO. 

Future research is needed to confirm our findings and examine the effects of prehabilitation on body composition during the preoperative period in these patients, as well as to elucidate the potential benefit of this approach in reducing postoperative complications.

## Figures and Tables

**Table 1:** Baseline characteristics of our series. ASA, American Society of Anesthesiologists; BMI, body mass index; CAD, coronary artery disease; CCI, Charlson comorbidity index; COPD, chronic obstructive pulmonary disease; ICU, intensive care unit; LVI, lymphovascular invasion; NLR, neutrophil-to-lymphocyte ratio; RT, radiotherapy; TURB, transurethral resection of the bladder.

Variable	Overall (*n* = 249)
Sex	
Male	203 (81.50%)
Female	46 (18.47%)
Age (yrs)	73.89 (10.03)
CCI	5.5 (4.94)
ASA class	
1	7 (2.90%)
2	138 (57.26%)
3	95 (39.42%)
4	1 (0.41%)
Current smoker	51 (20.48%)
Diabetes	41 (16.47%)
Hypertension	108 (43.20%)
Liver disease	16 (6.43%)
History of CAD	31 (12.40%)
COPD	37 (14.80%)
History of pelvic RT	13 (5.22%)
Neoadjuvant CT	27 (10.89%)
Prior rectal or abdominal surgery	72 (28.92%)
Creatinine levels	1.19 (0.91)
NLR	4.64 (6.05)
Serum total protein	6.65 (0.80)
Cholesterol levels	186.06 (39.37)
Triglyceride levels	124.28 (76.09)
Number of TURB	1.8 (1.47)
Carcinoma in situ	52 (21.67%)
Prior LVI	7 (3.18%)
Preoperative hydronephrosis	65 (26.21%)
Approach	
Open	132 (53.23%)
Laparoscopic	116 (46.77%)
Urinary diversion	
Ileal conduit	191 (80.93%)
Orthotopic neobladder	27 (11.44%)
Cutaneous ureterostomy	18 (7.63%)
ICU stay (days)	1.73 (1.69)
Mortality within 3 months after RC	7 (2.83%)
90-day complications	150 (60.24%)
Clavien-Dindo classification	
1	8 (5.33%)
2	96 (64.00%)
3a	2 (1.33%)
3b	24 (16.00%)
4	13 (8.67%)
5	7 (4.67%)
BMI	27.49 (4.16)
Obesity	53 (21.29)
Total muscle area (TMA)	138.65 (117.93–156.67)
Visceral fat area (VFA)	215.11 (149.44–286.14)
Subcutaneous fat area (SFA)	139.87 (98.56–190)
Total fat area (TFA)	359.295 (265.53–448.06)
Skeletal muscle mass index (SMI)	48.89 (42.47–54.36)
Sarcopenia	127 (52.48%)
Subcutaneous adipose tissue index (SATI)	49.74 (35.96–67.81)
Psoas muscle index (PMI)	5.80 (4.76–7.17)
Sarcopenic obesity (SO)	14 (5.62%)
Follow-up (months)	32.46 (16.43–53.1)

**Table 2:** Uni- and multivariate logistic regression analyses for postoperative complications in patients after RC. ASA, American Society of Anesthesiologists; BMI, body mass index; CAD, coronary artery disease; COPD, chronic obstructive pulmonary disease; NAC, neoadjuvant chemotherapy; NLR, neutrophil-to-lymphocyte ratio; RT, radiotherapy.

	Univariate		Multivariate (Final)	
Variable	OR (95% CI)	*p*	OR (95% CI)	*p*
Sex	1.35 (0.71–2.59)	0.35		
Age	0.32 (0.04–2.17)	0.11		
BMI	1.05 (0.96–1.16)	0.25		
Obesity	1.99 (1.03–3.87)	0.04 *		
ASA class	1.69 (1.04–2.74)	0.03 *		
Diabetes	0.96 (0.48–1.90)	0.91		
Hypertension	1.21 (0.73–2.02)	0.46		
History of CAD	1.78 (0.78–4.05)	0.16		
COPD	1.31 (0.63–2.71)	0.46		
History of pelvic RT	1.58 (0.47–5.28)	0.45		
NAC	0.98 (0.43–2.23)	0.97		
Prior rectal or abdominal surgery	1.85 (1.03–3.31)	0.03 *	2.56 (1.24–5.23)	0.011 *
Creatinine levels	1.06 (0.79–1.42)	0.67		
NLR	1.03 (0.97–1.10)	0.28		
Serum total protein	0.60 (0.39–0.92)	0.02 *	0.57 (0.36–0.88)	0.013 *
Cholesterol levels	0.99 (0.98–1.00)	0.25		
Triglyceride levels	0.99 (0.99–1.00)	0.26		
Preoperative hydronephrosis	1.22 (0.68–2.19)	0.49		
Total muscle area (TMA)	1.00 (0.99–1.00)	0.95		
Visceral fat area (VFA)	1.00 (0.99–1.00)	0.36		
Subcutaneous fat area (SFA)	1.00 (0.99–1.00)	0.08		
Total fat area (TFA)	1.00 (0.99–1.00)	0.41		
Skeletal muscle mass index (SMI)	1.00 (0.97–1.03)	0.78		
Sarcopenia	0.82 (0.49–1.38)	0.47		
Subcutaneous adipose tissue index (SATI)	1.00 (0.99–1.01)	0.12		
Psoas muscle index (PMI)	0.95 (0.84–1.06)	0.37		
Sarcopenic obesity (SO)	4.39 (1.02–20.10)	0.04 *	7.01 (1.06–37.05)	0.045 *

* denotes statistical significance.

**Table 3:** Association between sarcopenic obesity (SO) and different postoperative complications.

Variable	Overall	SO (*n* = 14)	Non-SO (*n* = 235)	*p*
Anastomotic leak (bowel)	6 (2.76%)	0%	6 (2.94)	0.53
Urinary leakage	7 (2.81%)	0%	7 (2.98%)	1.00
Evisceration and eventration	9 (4.15%)	2 (15.38%)	7 (3.43%)	0.036 *
Intra-abdominal complication	65 (29.95%)	3 (23.08%)	62 (30.39%)	0.75

* denotes statistical significance.

**Table 4:** Uni- and multivariate Cox regression analyses of risk factors for OS, CSS, and PFS after RC. ASA, American Society of Anesthesiologists; BMI, body mass index; CKD, chronic kidney disease; LVI, lymphovascular invasion; NAC, neoadjuvant chemotherapy; NLR, neutrophil to lymphocyte ratio; RT, radiotherapy; TURB, transurethral resection of the bladder.

	Overall Survival (OS)				Cancer-Specific Survival (CSS)				Progression-Free Survival (PFS)			
	Univariate		Multivariate (Final)		Univariate		Multivariate (Final)		Univariate		Multivariate (Final)	
Variable	HR (95% CI)	*p*	HR (95% CI)	*p*	HR (95% CI)	*p*	HR (95% CI)	*p*	HR (95% CI)	*p*	HR (95% CI)	*p*
Sex	1.18 (0.70–2.00)	0.51			0.98 (0.52–1.85)	0.96			0.85 (0.51–1.42)	0.54		
Age	1.06 (1.03–1.08)	0.000 *	1.04 (1.02–1.06)	0.001 *	1.06 (1.03–1.10)	0.000 *	1.07 (1.03–1.11)	0.000 *	1.03 (1.01–1.06)	0.001 *	1.02 (1.01–1.04)	0.04 *
BMI	0.94 (0.87–1.02)	0.173			0.97 (0.88–1.07)	0.61			1.02 (0.95–1.10)	0.54		
Obesity	1.01 (0.62–1.63)	0.969			1.00 (0.54–1.85)	0.98			1.29 (0.81–2.07)	0.28		
ASA class	1.88 (1.29–2.72)	0.001 *			1.49 (0.94–2.39)	0.09			1.16 (0.80–1.70)	0.42		
NAC	0.44 (0.18–1.09)	0.07			0.27 (0.06–1.10)	0.06			0.75 (0.36–1.56)	0.45		
Prior rectal or abdominal surgery	1.41 (0.93–2.13)	0.09			1.30 (0.77–2.21)	0.31			1.39 (0.91–2.15)	0.13		
Creatinine levels	1.19 (1.01–1.40)	0.033 *			1.13 (0.89–1.43)	0.28			1.13 (0.95–1.35)	0.15		
NLR	1.02 (0.99–1.05)	0.101			1.03 (1.01–1.06)	0.016 *			1.03 (1.01–1.05)	0.013 *		
Serum total protein	0.82 (0.63–1.07)	0.15			0.82 (0.59–1.14)	0.25			0.88 (0.67–1.17)	0.41		
Number of TURB	0.97 (0.84–1.12)	0.67			0.75 (0.57–0.99)	0.04 *			0.91 (0.77–1.07)	0.28		
Prior intravesical therapy	0.95 (0.58–1.53)	0.82			0.62 (0.29–1.29)	0.20			0.75 (0.45–1.28)	0.30		
Carcinoma in situ (CIS)	0.72 (0.21–1.20)	0.21			0.64 (0.32–1.26)	0.20			0.44 (0.24–0.84)	0.013 *		
Prior LVI	3.33 (1.45–7.66)	0.005 *			3.56 (1.28–9.90)	0.015 *	2.87 (1.01–8.19)	0.048 *	1.97 (0.62–6.29)	0.25		
Preoperative hydronephrosis	1.77 (1.16–2.71)	0.007 *			1.67 (0.97–2.87)	0.06			2.05 (1.32–3.17)	0.001 *		
CKD after RC	1.35 (0.91–2.02)	0.13			1.28 (0.77–2.13)	0.32			1.59 (1.05–2.40)	0.026 *		
Postoperative hydronephrosis	1.45 (0.96–2.18)	0.07			1.37 (0.82–2.31)	0.22			1.25 (0.82–1.89)	0.28		
CIS on RC specimen	0.54 (0.35–0.83)	0.005 *			0.40 (0.23–0.73)	0.002 *	0.45 (0.24–0.84)	0.013 *	0.56 (0.36–0.88)	0.011 *		
LVI on RC specimen	3.44 (2.19–5.40)	0.000 *	1.65 (1.03–2.65)	0.039 *	3.81 (2.18–6.65)	0.000 *	1.97 (1.06–3.64)	0.030 *	3.51 (2.19–5.59)	0.000 *		
>T1	5.77 (3.26–10.21)	0.000 *	3.78 (2.06–6.92)	0.000 *	10.39 (4.14–26.05)	0.000 *			5.71 (3.16–10.31)	0.000 *	3.99 (2.15–7.41)	0.000 *
N+	3.02 (2.01–4.56)	0.000 *	1.76 (1.14–2.71)	0.01 *	5.51 (3.29–9.21)	0.000 *	4.99 (2.83–8.78)	0.000 *	3.45 (2.34–5.08)	0.000 *	2.37 (1.57–3.58)	0.000 *
Surgical margins	2.67 (1.48–4.81)	0.001 *			3.79 (1.96–7.33)	0.000 *			2.63 (1.43–4.85)	0.002 *		
Total muscle area (TMA)	0.99 (0.98–1.01)	0.11			0.99 (0.98–1.00)	0.19			0.99 (0.98–1.00)	0.35		
Visceral fat area (VFA)	0.99 (0.99–1.00)	0.19			0.99 (0.99–1.00)	0.23			0.99 (0.99–1.00)	0.64		
Subcutaneous fat area (SFA)	0.995 (0.92–0.99)	0.01 *			0.99 (0.99–1.00)	0.17			0.99 (0.99–1.00)	0.61		
Total fat area (TFA)	0.99 (0.99–1.00)	0.19			0.99 (0.99–1.00)	0.33			1.00 (0.99–1.00)	0.96		
Skeletal muscle mass index (SMI)	0.99 (0.97–1.01)	0.39			0.99 (0.97–1.02)	0.90			1.00 (0.98–1.02)	0.86		
Sarcopenia	1.42 (0.95–2.12)	0.08			1.50 (0.89–2.52)	0.12			1.03 (0.68–1.55)	0.88		
Subcutaneous adipose tissue index (SATI)	0.99 (0.98–0.99)	0.03 *			0.99 (0.98–1.01)	0.40			0.99 (0.99–1.00)	0.81		
Psoas muscle index (PMI)	0.96 (0.88–1.04)	0.38			0.97 (0.90–1.05)	0.57			0.97 (0.91–1.04)	0.41		
Sarcopenic obesity (SO)	2.01 (1.01–3.99)	0.04 *			1.77 (0.71–4.44)	0.21			1.33 (0.58–3.05)	0.49		

* denotes statistical significance.

## Data Availability

The anonymized datasets generated during and/or analyzed during the current study are available from the corresponding author on reasonable request.

## Supplementary Materials

The following supporting information can be downloaded at: https://www.mdpi.com/article/10.3390/cancers17162669/s1, Table S1. The relationship between body composition phenotypes and tumor stage, lymph node involvement (N+), and history of neoadjuvant chemotherapy (NAC). Table S2. Studies addressing the predictive value of sarcopenia and SO on postoperative outcomes after RC. A search on PubMed (MEDLINE) was conducted using the terms “Sarcopenia”, “Cystectomy”, and “Bladder cancer”. Figure S1. Pre- and postprocessing steps to assess body composition parameters. Figure S2. Incidence of postoperative complications among the different patient groups (SO vs. non-SO). In the non-SO group, complications were reported in 57.69% of patients, while the rate was 85.7% among SO patients. Figure S3. Kaplan–Meier survival curves stratified by the presence of SO in the entire cohort for OS (A), CSS (B), and PFS (C). There were no statistically significant differences in survival curves between the groups.

## Abbreviations

ASA	American Society of Anesthesiologists
BMI	Body mass index
CCI	Charlson Comorbidity Index
CT	Computed tomography
CSS	Cancer-specific survival
ICU	Intensive care unit
OS	Overall survival
PFS	Progression-free survival
RC	Radical cystectomy
SMI	Skeletal muscle index
SO	Sarcopenic obesity
