# Supplementary material for: Impact of Preoperative CT-Diagnosed Sarcopenic Obesity on Outcomes After Radical Cystectomy for Bladder Cancer
Source: Cancers (Basel). 2025 Aug 15;17(16):2669. doi: 10.3390/cancers17162669 (PMC12384084; doi:10.3390/cancers17162669)
Supplement: Supplementary file 1 [file cancers-17-02669-s001.zip › cancers-3739763-supplementary.pdf]

cancers MDPI

Impact of Preoperative CT-Diagnosed Sarcopenic Obesity on Outcomes After Radical Cystectomy for Bladder Cancer

Alberto Artiles Medina, Mariam Bajawi Carretero, Enrique López Pérez, Sara Garach Fernández, David López Curtis, Leyre Elías Pascual, José Daniel Subiela, Javier Soto Pérez-Olivares, Catalina Nieto Góngora, Fernando González Tello, Irene de la Parra Sánchez, César Mínguez Ojeda, Victoria Gómez Dos Santos and Francisco Javier Burgos Revilla

Supplementary Table S1. The relationship between body composition phenotypes and tumor stage, lymph node involvement (N+), and history of neoadjuvant chemotherapy (NAC).

Variable	Obese (n=53)/non-obese	Sarcopenic (n=127)/non-sarcopenic	SO (n=14)/non-SO
Tumor stage >T2	21/96 (p=0.27)	64/50 (p=0.26)	8/108 (p=0.27)
Lymph node involvement (N+)	12/46 (p=0.92)	37/19 (p=0.06)	5/53 (p=0.19)
NAC	5/22 (p=0.69)	18/8 (p=0.08)	0/27 (p=0.18)

Supplementary Table S2. Studies addressing the predictive value of sarcopenia and SO with respect to postoperative outcomes after RC. A search on PubMed (MEDLINE) was conducted using the terms "Sarcopenia", "Cystectomy", and "Bladder cancer". BMI, body mass index; CSS, cancer-specific survival; NR, not recorded; OS, overall survival; PMI, psoas major muscle index; SMI, skeletal muscle index; SO, sarcopenic obesity; TPI, total psoas index.

Author (year)	n	Prevalence of sarcopenia	Definition of sarcopenia	Prevalence of SO	Predictive value of sarcopenia for postoperative complications	Predictive value of sarcopenia for survival outcomes	Predictive value of SO for postoperative complications	Predictive value of SO for survival outcomes
Smith et al. (2014) [23]	200	77/200 (38.5%)	Total psoas area: cutoff of 523 cm²/m² (AUC 0.70)	NR	YES (major complications in women)	NO (2-year survival)	NR	NR
Psutka et al. (2014) [24]	205	141 (68.8%)	SMI of <55 cm²/m² for men and <39 cm²/m² for women	NR	NR	YES (increased CSS and all-cause mortality)	NR	NR
Psutka et al. (2014) [15]	207	NR	NR	34 (16.4%)	NR	NR	NR	NO (SO did not independently predict cancer-specific mortality or all-cause mortality)
Hirasawa et al. (2016) [25]	136	65 (47.8%)	SMI of <43 cm²/m² for men with BMI <25 kg/m², SMI <53 cm²/m² for men with BMI ≥25 kg/m², and SMI <41 cm²/m² for women (Martin et al.)	NR	NR	YES (shorter CSS)	NR	NR
Saitoh-Maeda et	78	NR	NR	NR	YES (longer hospitalization)	YES (worse OS in men	NR	NR

al. (2017) [26]						with a PMI <400)		
Mayr et al. (2018) [13]	500	189 (37.8%)	SMI of <43 cm²/m² for men with BMI <25 kg/m², SMI <53 cm²/m² for men with BMI ≥25 kg/m², and SMI <41 cm²/m² for women (Martin et al.)	18/189	NR	YES (both increased all-cause mortality and CS mortality)	NR	NR
Mavuduru et al. (2019) [14]	54	17 (30.9%)	SMI of < 43 cm²/m² for men with BMI <25 kg/m², SMI <53 cm²/m² for men with BMI ≥25 kg/m², and SMI <41 cm²/ m² for women	8/17 (BMI >25 overweight-obese)	NO	NR	NR	NR
Fraisse et al. (2020) [27]	146	67/146 (45.9%)	Two ways: either by SMI without muscle mass adjustment or according to the definition by Martin et al. based on gender and patient BMI, then called "adjusted sarcopenia"	NR	NO	NO	NR	NR
Mao et al. (2020) [28]	200	67 (33.5%)	TPI <385 mm²/m² for female patients or TPI <545 mm²/m² for male patients	NR	NR	YES (protective, independent risk predictor of OS and DFS)	NR	NR
Yamashita et al. (2021) [29]	123	48 (39%	SMI <40.8 cm²/m² for men and SMI <34.9 cm²/m² for women	NR	NR	YES (poor cancer-specific survival)	NR	NR
Stangl-Kremser et al. (2021) [30]	441	143 (32.4%)	PMI ≤7.4 cm²/m² and female patients with a PMI ≤5.2 cm²/m² (L4 TPA measurements)	NR	YES (30-day complications)	NO	NR	NR
Ying et al. (2021) [31]	292	NR	NR	NR	NO	YES (OS)	NR	NR
Engelmann et al. (2023) [32]	657	340 (52.8%)	Several definitions*	NR	NR	YES (shorter OS and CSS)	NR	NR
Erdik et al. (2023) [33]	84	45 (53.6%)	SMI <43 cm²/m² for men with BMI <25 kg/m², SMI <53 cm²/m² for men with BMI ≥25 kg/m², and SMI <41 cm²/m² for women (Martin et al.)	NR	NR	YES (increased CSS and overall mortality)	NR	NR
Lee et al. (2024) [34]	528	37.9%	SMI <43 cm²/m² for men with BMI <25 kg/m², SMI <53 cm²/m² for men with BMI ≥25 kg/m², and SMI <41 cm²/m² for women (Martin et al.)	NR	NR	YES (CSS)	NR	NR
Sharma et al. (2025) [35]	843	NR	NR They analyzed SMI (SMA in cm²/height in m²) and fat mass index (total fat area in cm²/height in m², FMI)	NR	YES (a higher preoperative SMI was associated with lower odds of major complications,	NR	NR	NR

	SMI cutoff points: 38.6 (30.7) and 36.9 (28.9) for men (women)	while higher intramuscular adipose was associated with higher odds of major complications)	

* **SMI (Martin [20]):** Males: SMI <43 cm^2/m^2 if BMI <25 kg/m^2 or SMI <53 cm^2/m^2 if BMI ≥25 kg/m^2; females: SMI <41 cm^2/m^2. **SMI (Caan [36])**: Males: SMI <52.3 cm^2/m^2 if BMI <30 kg/m^2 or SMI <54.3 cm^2/m^2 if BMI ≥30 kg/m^2; females: SMI <38.6 cm^2/m^2 if BMI <30 kg/m^2 or SMI <46.6 cm^2/m^2 if BMI ≥30 kg/m^2. **SMI (Prado [19])**: Males: SMI ≤52.4 cm^2/m^2; females: SMI ≤38.5 cm^2/m^2. **SMI (Lancet Oncology Consensus [37])**: Males: SMI <55 cm^2/m^2; females: SMI <39 cm^2/m^2.

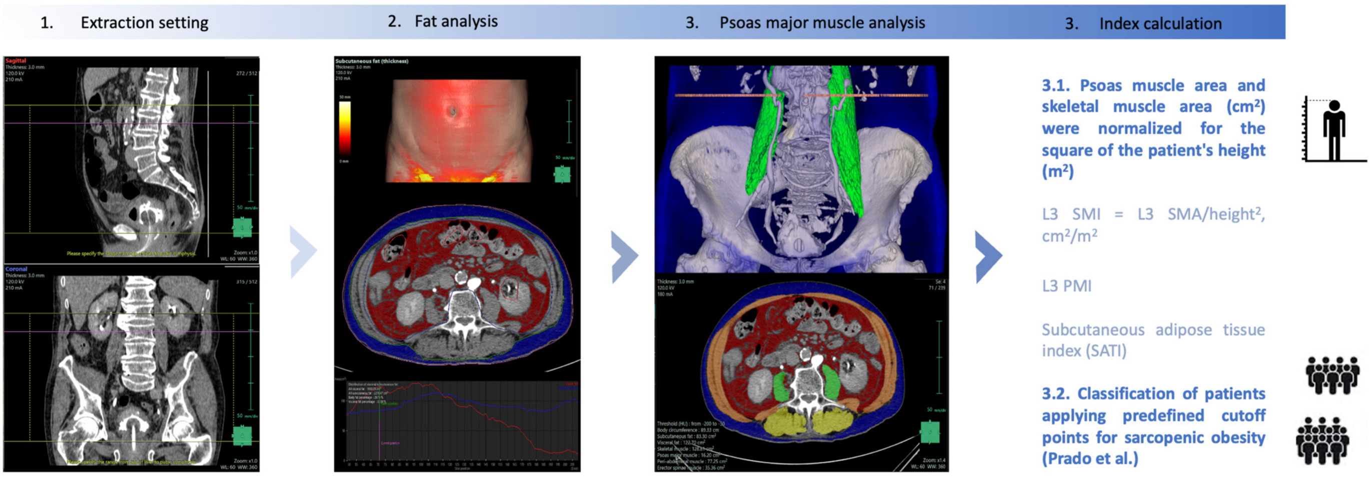

Supplementary Figure S1. Pre- and postprocessing steps to assess body composition parameters.

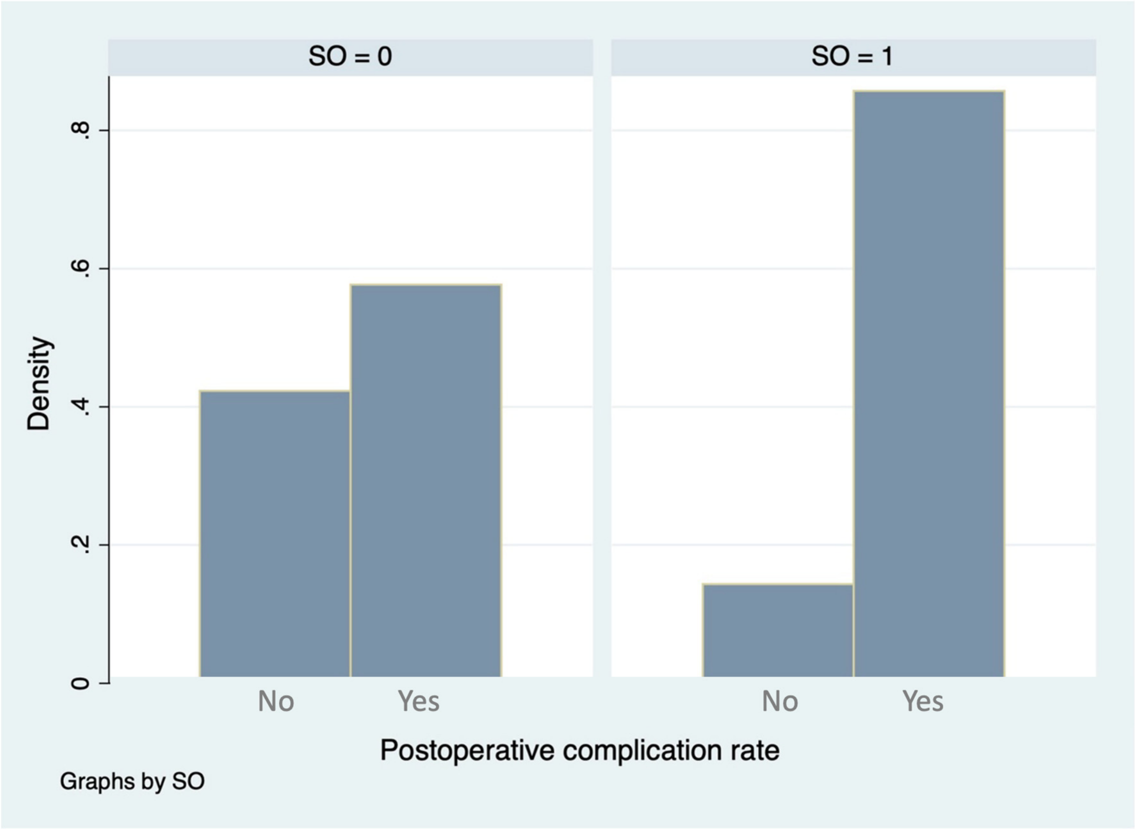

Supplementary Figure S2. Incidence of postoperative complications among the different patient groups (SO vs non-SO). In the non-SO group, complications were reported in 57.69% of patients, while the rate was 85.7% among SO patients.

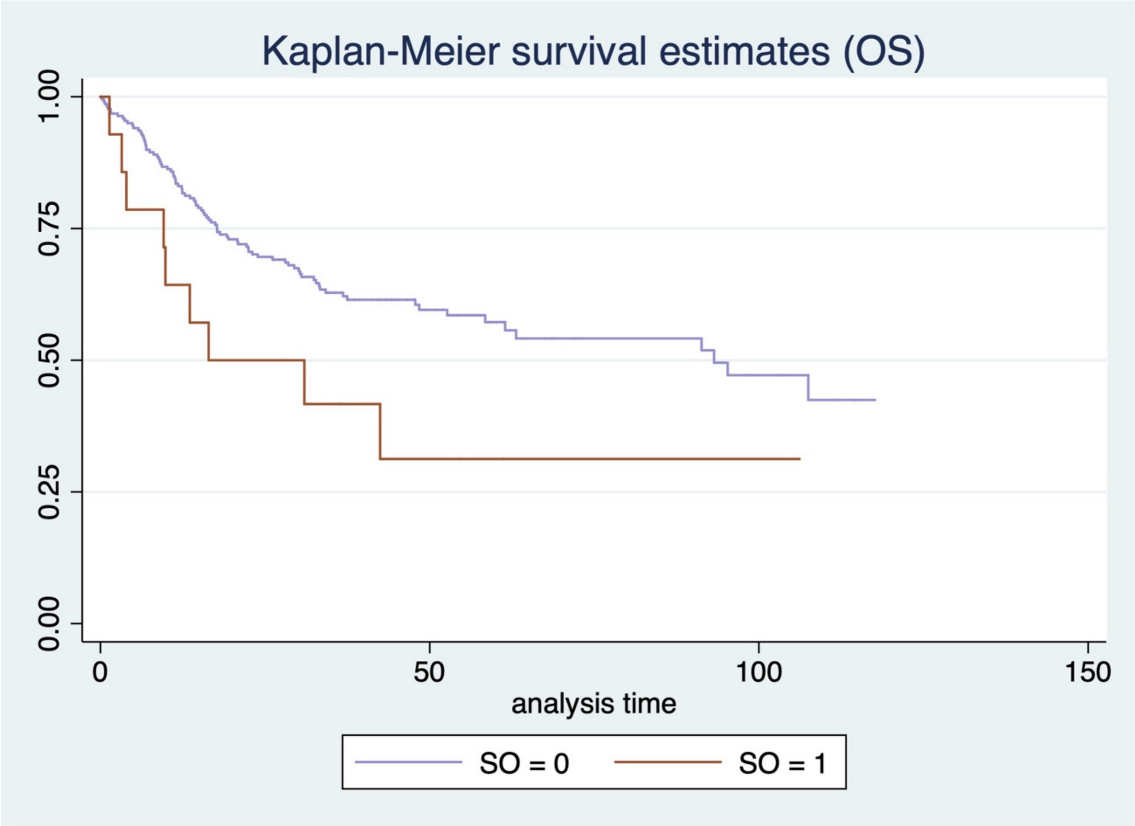

Kaplan-Meier survival estimates (OS)
1.00
0.75
0.50
0.25
0.00
0
50
100
150
analysis time
SO = 0
SO = 1

(A)

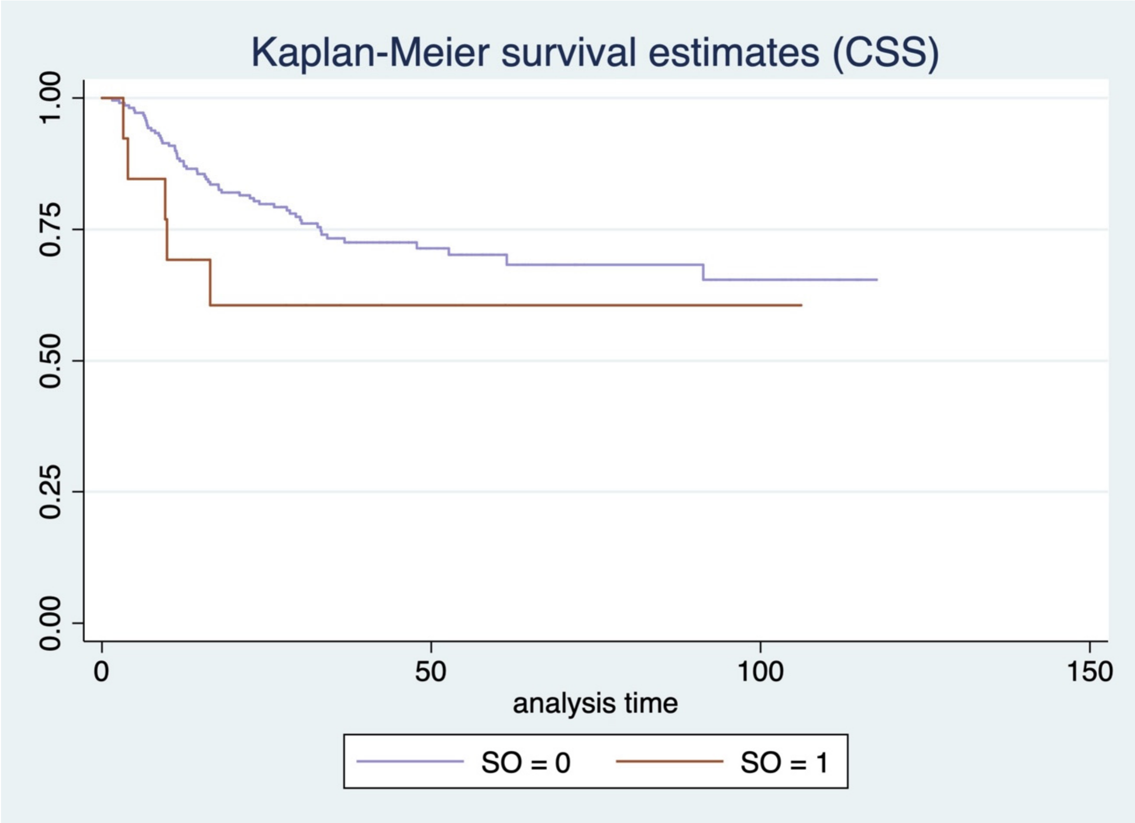

Kaplan-Meier survival estimates (CSS)
1.00
0.75
0.50
0.25
0.00
0
50
100
150
analysis time
SO = 0
SO = 1

(B)

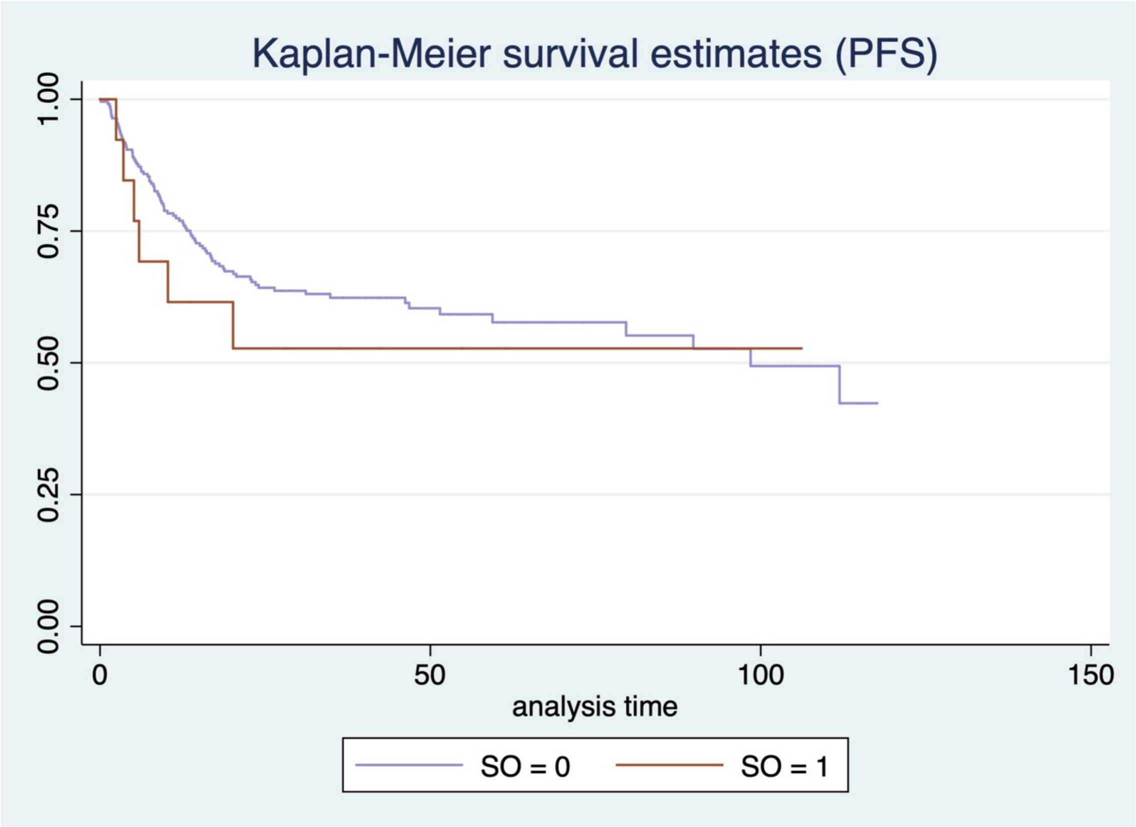

(C)

Supplementary Figure S3. Kaplan–Meier survival curves stratified by the presence of SO in the entire cohort for OS (**A**), CSS (**B**), and PFS (**C**). There were no statistically significant differences in survival curves between the groups.
